# High-dose chemotherapy and autologous stem cell transplantation of patients with multiple myeloma in an outpatient setting

**DOI:** 10.1186/s12885-017-3137-4

**Published:** 2017-02-22

**Authors:** Katharina Lisenko, Sandra Sauer, Thomas Bruckner, Gerlinde Egerer, Hartmut Goldschmidt, Jens Hillengass, Johann W. Schmier, Sofia Shah, Mathias Witzens-Harig, Anthony D. Ho, Patrick Wuchter

**Affiliations:** 1 Department of Medicine V, Heidelberg University, Im Neuenheimer Feld 410, 69120 Heidelberg, Germany; 2 Institute of Medical Biometry und Informatics, Heidelberg University, Marsilius Arkaden 130.3, 69120 Heidelberg, Germany; 3 National Center for Tumor Diseases Heidelberg (NCT), Im Neuenheimer Feld 460, 69120 Heidelberg, Germany; 4 Institute of Transfusion Medicine and Immunology, German Red Cross Blood Service Baden-Württemberg—Hessen, Medical Faculty Mannheim, Heidelberg University, Friedrich-Ebert-Straße 107, 68167 Mannheim, Germany

**Keywords:** Multiple myeloma, Autologous blood stem cell transplantation, High-dose chemotherapy, Outpatient setting, Outpatient supportive care

## Abstract

**Background:**

High-dose (HD) chemotherapy with melphalan and autologous blood stem cell transplantation (ABSCT) for treatment of symptomatic multiple myeloma (MM) on an outpatient basis has been well established in the USA and Canada, whereas in Germany and Western Europe an inpatient setting is the current standard. We report on a German single-centre program to offer the procedure on an outpatient basis to selected patients.

**Methods:**

Major requirements included: patients had to have family and/or other caregivers, had to be able to reach the hospital within 45 min and have an ECOG performance score of 0–1. Patients with severe co-morbidities were not included.

**Results:**

From September 2012 until April 2016, 21 patients with MM stage IIIA were enrolled. All engrafted within the expected time range (median 14 days), and no severe adverse events occurred. 14 patients (67%) had an episode of neutropenic fever and blood cultures were positive in 4 patients (19%). Although rather liberal criteria for hospital admission were applied, 14 patients (67%) were treated entirely on an outpatient basis.

**Conclusions:**

HD chemotherapy and ABSCT on an outpatient basis is safe and feasible if it is conducted in an elaborate surveillance program. The feedback from patients was very positive, thus encouraging further expansion of the program.

## Background

High-dose (HD) chemotherapy and autologous blood stem cell transplantation (ABSCT) is a standard of care in transplant eligible patients with multiple myeloma (MM) and a variety of malignant diseases [1–4]. Initially established as a single ABSCT in newly diagnosed MM, subsequent trials have shown the benefit of a tandem ABSCT in overall survival, particularly in those patients who do not reach at least a very good partial remission after the first autograft [5–8]. Moreover, HD chemotherapy and ABSCT is also an effective treatment option for relapse MM patients [9–11].

MM patients undergoing HD chemotherapy and ABSCT have traditionally been admitted to the hospital for several weeks. With the increasing ABSCT experience of the transplanting centres, the patients’ wish for a shorter stay in the hospital, increasing number of nosocomial infections and growing economic pressure, particularly in view of increasing absolute numbers of ABSCTs in Europe during the last decade [12], there is a clear trend towards outpatient treatment. The experience from performing HD chemotherapy and ABSCT in an outpatient setting in the late 1990s has indicated a high degree of safety, feasibility, cost saving and patient satisfaction [13–16]. Although it has been well established in the United States of America [17–19] and Canada [20, 21], only single reports on outpatient HD chemotherapy and ABSCT in Europe are available [22, 23]. To the best of our knowledge, the outpatient treatment option has not been established as a routine in a transplant centre in Germany so far.

Since 2012, we have performed HD chemotherapy and ABSCT on an outpatient basis in individually selected MM patients within an elaborated program. Currently, this outpatient ABSCT program is being extended to a higher case number, and up to 2 patients undergo this treatment in parallel at a time. This report summarizes our experience of 21 ABSCTs performed on an outpatient basis. The aim of this retrospective study is to demonstrate our approach and analyse the safety and efficacy of the program.

## Methods

### Study design and data collection

A retrospective single-centre analysis of MM patients (*n* = 21) who underwent HD melphalan chemotherapy and ABSCT as an outpatient between September 2012 and February 2016 at our University Hospital outpatient clinic was performed. Clinical parameters (gender, age, Eastern Cooperative Oncology Group (ECOG) score), disease stage at first diagnosis according to Salmon and Durie, type of monoclonal protein, modality of induction and mobilization therapy, peripheral blood stem cell (PBSC) collection result, remission status pre and post ABSCT, transplanted CD34+ cell number, haematological reconstitution data, toxicities and supportive interventions were assessed retrospectively.

To identify potential factors predicting the need for inpatient admission and illustrate the possible differences in toxicities and haematological reconstitution, patients were retrospectively grouped according to the necessity of hospital admission (hereafter referred to as “outpatients” and “temporary inpatients”). Retrospective data analysis was approved by the Ethics Committee of the Medical Faculty, Heidelberg University. Patients’ informed written consent was obtained.

### Inclusion and exclusion criteria and safety issues

The following inclusion criteria were defined: general transplantation eligibility, age 18–70 years, ECOG performance status 0–1, implanted port catheter system or excellent peripheral vein conditions, availability of an accompanying care-taking person, availability by cell phone, transport distance from home or hotel to the outpatient clinic of ≤45 min, patient’s compliance with the given instructions and patient’s informed consent.

The exclusion criteria were defined as follows: light-chain amyloidosis, detection of antibodies to human leukocyte antigens (HLA) and/or insufficient platelet increase after platelet transfusion, insurmountable language barrier, medical complications during induction or mobilization therapy and severe comorbidities like cardiac or renal insufficiency.

There was an intention to treat all of the patients on an outpatient basis in our outpatient clinic. If indicated, a hospital admission could be arranged instantly, and patients could contact a haematologist by phone at any time. All of the patients received a detailed information brochure regarding the prevention of infection, body care, oral hygiene, diet and physical activity during aplasia.

### HD chemotherapy and ABSCT

Indication and eligibility for HD melphalan and ABSCT were determined by the treating physician. All of the patients received HD melphalan (100 mg/m^2^, day -3 and day -2, 1 h infusion) as the conditioning regimen. The melphalan dosage was reduced by 50% due to comorbidities in one patient with refractory myeloma who had already undergone three previous courses of HD melphalan chemotherapy and ABCST. A minimum of 2.0 × 10^6^ CD34^+^ cells/kg patient’s body weight was re-infused in all cases on day 0 using standard supportive therapy (500 mg acetaminophen p.o., 2 mg clemastine intravenous (i.v.), and 10 mg dihydrocodeine p.o.). No growth factors were used post-transplantation.

### Monitoring and Supportive Care

#### Monitoring

During patient monitoring visits, clinical examination, vital signs assessment (blood pressure, heart rate, body temperature, weight) and laboratory testing (blood count, electrolytes, creatinine, liver values, coagulation status and C-reactive protein) were performed daily, including weekends. All visits as well as any treatment took place in the outpatient clinic in a specified area and with staff previously introduced to the patients in order to avoid any stay in the waiting area to reduce the risk of infection. Daily visits were continued until recovery of leucocytes >1.0 × 10^9^/L, neutrophils >0.5 × 10^9^/L and platelets >50 × 10^9^/L in the absence of any signs of infection.

#### Antiemetic prophylaxis

Compared to an antiemetic prophylaxis given in the inpatient setting [24], an intensified oral supportive medication for preventing chemotherapy-induced nausea and vomiting was administered: dexamethasone 2 to 4 mg day -3 and dexamethasone 1 to 2 mg day -2 to day -1, granisetron 2 mg days -3 to day +4, aprepitant 125 mg day -3, aprepitant 80 mg day -2 to day +2. Dimenhydrinate and/or metoclopramide p.o. were prescribed to the patients as home medication, if required. Moreover, pantoprazole 40 mg p.o. was administered once daily.

#### Hydration and prophylaxis of stomatitis

For all patients, 1 to 2 L of 0.9% saline solution and, depending on the serum potassium level, 10–30 mval potassium chloride were administered by i.v. daily. To prevent stomatitis, patients were strongly recommended to rinse the mouth with Caphosol® (calcium-phosphate solution) at least once per hour during their stay at the outpatient clinic and at home.

Non-steroidal anti-rheumatics were avoided due to unintended fever suppression, but opioid analgesics were used for pain management, e.g. in case of stomatitis.

#### Antiviral and antibiotic prophylaxis and treatment

Patients received daily ciprofloxacin 2×500 mg per os (p.o.) until haematological reconstitution and aciclovir 2×400 mg p.o. for 6 months after ABSCT. In case of fever (>38.3 °C), an empirical antibiotic treatment with 1 g ertapenem i.v. per 24 h was initiated. Blood cultures were obtained and further diagnostic tests including imaging techniques were performed if necessary. At the discretion of the treating physician, the empirical i.v. antibiotic therapy was initiated in some cases at subfebrile temperatures when C-reactive protein (CRP) elevation was observed. In case of persisting fever >72 h, antibiotic therapy was substituted by i.v. 3x piperacillin 4 g/tazobactam 0.5 g per 24 h, and the patient was admitted to the hospital. Intravenous antibiotic therapy was continued until the fifth day without fever or haematological reconstitution.

### Criteria for inpatient admission and discharge

Rounds were conducted daily, and the patient’s clinical status was evaluated by the treating physician. In alignment with previously published policies of other centres [15], we pursued a rather liberal strategy for hospitalization of outpatients, primarily based on clinical parameters. Patients were admitted to the hospital in case of fever persisting for more than 72 h. Further criteria for inpatient admission were ECOG score >2, pneumonia, sepsis, uncontrolled pain, diarrhoea and an indication for parenteral nutrition in case of grade 3 stomatitis or nausea and vomiting. A discharge from hospital and further treatment again as an outpatient was possible, depending on the patient’s clinical status.

### Assessment of haematological reconstitution and remission status

After ABSCT, blood count was performed on a daily basis until platelet and leucocyte/neutrophil engraftment. Platelet engraftment was defined as the first of 3 consecutive days on which platelets reached 20 × 10^9^/L without platelet transfusion. Because the platelet count did not fall below 20 × 10^9^/L or a platelet transfusion was necessary in some patients, we also assessed days until platelets ≥50 × 10^9^/L as a second variable for platelet engraftment. Leucocyte engraftment was defined by a leucocyte count of ≥1.0 × 10^9^/L. Days with leucocytes <1.0 × 10^9^/L were recorded as days in aplasia. Neutrophil recovery was defined as the first of 3 days on which neutrophils reached 0.5 × 10^9^/L. The remission status was assessed according to international uniform response criteria for MM [25].

### Assessment of patient satisfaction

Patients’ satisfaction was assessed using a structured questionnaire after hematologic reconstitution when daily monitoring at the outpatient clinic was discontinued. The patients were asked to give marks ranging from 1 (very good) to 6 (unsatisfactory) for the medical care provided by physicians and nurses and for the treatment at the outpatient clinic as a whole. They were also asked to determine whether they would, if indicated, undergo further HD chemotherapy and ABSCT in the outpatient setting again.

### Statistical Analysis

Descriptive statistics and comparison between groups were performed using R studio 7.6. Data are given as absolute numbers and percentage and, if not otherwise stated, the median and range. For the comparison of categorical variables, Fisher’s Exact test in case of 2 × 2 contingency tables or its Freeman-Halton extension in case of 2 × >2 contingency tables was used. To identify differences among groups in case of continuous variables, a Wilcoxon-Mann-Whitney test was performed. Leucocyte, neutrophil and platelet recovery over time was calculated and plotted using Kaplan-Meier survival analysis. To calculate the differences between the engraftment curves, a log-rank test was used. A *P*-value <0.05 was considered statistically significant.

## Results

### Patients’ characteristics

Overall, 21 MM patients were identified as candidates for an outpatient treatment, and HD chemotherapy and ABSCT was initiated in our outpatient clinic. In 14 cases (67%), therapy was performed completely on an outpatient basis. In 7 patients (33%), hospital admission and at least temporary inpatient treatment were indicated. Patients were grouped according to the necessity of hospital admission (“outpatients” vs. “temporary inpatients”).

More than twice as many male than female patients (*n* = 15 vs. *n* = 6) were intended to be treated on an outpatient basis. ECOG performance status prior to HD chemotherapy and ABSCT was 0 in 20 (95%) and 1 in 1 (5%) patients. All patients had an available accompanying person throughout the treatment period, except for one patient who suddenly and unexpectedly no longer had a care-giving family member available. Almost all of the patients (*n* = 19, 90%) had a central port catheter system. The majority of the patients had received bortezomib, doxorubicin, dexamethasone (PAD) or bortezomib, cyclophosphamide, dexamethasone (VCD) as induction therapy. Virtually all of the patients (*n* = 20, 95%) had received cyclophosphamide, doxorubicin, dexamethasone (CAD) for stem cell mobilization. A median PBSC stem cell collection result of 9.7 (range 7.4–24.8) and 13.7 (range 9.1–23.0) CD34+ cells x10^6^/kg was noted in out- and inpatients, respectively. Further details are shown in Table 1. No significant differences were found in the patients’ characteristics between outpatient and temporary inpatient cases.

**Table 1:** Patients’ characteristics

Parameters	Overall cohort	Outpatient treatment	Hospital admission	*P*-value
Patient number, n	21	14	7	/
Gender, n (%)				0.61
Male	15 (71)	9 (64)	6 (86)	
Female	6 (29)	5 (36)	1 (14)	
ECOG, n (%)				
0–1	21 (100)	14 (100)	7 (100)	1.00
Social conditions, n (%)	besser			0.33
Accompanying person available	20 (95)	14 (100)	6 (86)	
Single	1 (5)	0 (0)	1 (14)	
Implanted port catheter, n (%)				0.53
yes	19 (90)	12 (86)	7 (100)	
no	2 (10)	2 (14)	0 (0)	
Diagnosis of MM				
Age at first diagnosis, years	58 (43–67)	57 (43–67)	59 (47–66)	0.85
Stage at first diagnosis III, n (%)				1.00
A	21 (100)	14 (100)	7 (100)	
B	0 (0)	0 (0)	0 (0)	
Heavy chain type, n (%)				0.34
IgG	11 (52)	9 (64)	2 (29)	
IgA	9 (43)	5 (36)	4 (57)	
Light chain only	1 (5)	0 (0)	1 (14)	0.33
Light chain type, n (%)				1.00
κ	13 (62)	9 (64)	4 (57)	
λ	8 (38)	5 (36)	3 (43)	
Induction therapy, n (%)				0.61
PAD	10 (48)	5 (36)	5 (71)	
VAD	2 (10)	2 (14)	0 (0)	
VCD	8 (38)	6 (43)	2 (29)	
Other	1 (5)	1 (7)	0 (0)	
Mobilization therapy, n (%)				1.00
1xCAD	20 (95)	13 (93)	7 (100)	
Other	1 (5)	1 (7)	0 (0)	
PBSC collection				
Age at PBSC collection, years	59 (44–67)	57 (44–67)	59 (47–67)	0.79
Collected CD34+ cells × 10^6^/kg	11.8 (7.4–24.8)	9.7 (7.4–24.8)	13.7 (9.1–23.0)	0.20

*CAD* cyclophosphamide, doxorubicin, dexamethasone, *ECOG* Eastern Cooperative Oncology Group, *MM* multiple myeloma, *PAD* bortezomib, doxorubicin, dexamethasone, *PBSC* peripheral blood stem cells, *VAD* vincristine, doxorubicin, dexamethasone, *VCD* bortezomib, cyclophosphamide, dexamethasone. Unless otherwise indicated, data are given as medians (range)

### HD chemotherapy and ABSCT

The majority of patients received HD chemotherapy and ABSCT as a first-line treatment (*n* = 15, 71%). 6 patients (29%) received an autologous transplant as part of a salvage therapy regimen. 3 patients underwent a second course of HD chemotherapy and ABSCT in an outpatient setting as consolidation therapy or in case of relapse after 3–28 months. The median age at ABSCT was 59 (51–70) and 62 (51–67) years in out- and inpatients, respectively. All of the patients received HD melphalan. In one case, the melphalan dosage was reduced to 50% as an individual decision in a heavily pretreated patient, as described above. The remission status is summarized in Table 2.

**Table 2:** HD chemotherapy and transplant characteristics

Parameters	Overall cohort
ABSCT number, n	21
Age at ABSCT, years	59 (51–70)
Therapy line, n (%)	
First line therapy	15 (71)
Salvage therapy	6 (29)
Remission prior HD/ABSCT, (%)	
CR	0 (0)
nCR	5 (24)
VGPR	6 (29)
PR	8 (38)
MR	0 (0)
SD	1 (5)
PD	1 (5)
HD chemotherapy	
HD melphalan n, (%)	20 (95)
Dose modification, n (%)	1 (5)
ABSCT	
Transplanted CD34+ cells × 10^6^/kg	3.3 (2.1–6.5)
PBSC storage duration, months	2 (0–144)
Remission post HD and ABSCT	
CR	2 (10)
nCR	8 (38)
VGPR	3 (14)
PR	7 (33)
MR	0 (0)
SD	1 (5)
PD	0 (0)

*ABSCT*autologous blood stem cell transplantation, (*n*)*CR* (near) complete remission, *HD* high dose, *MR* minimal response, *PBSC* peripheral blood stem cells, *PD* progressive disease, *PR* partial remission, *SD* stable disease, *VGPR* very good partial remission. Unless otherwise indicated, data are given as medians (range)

### Post-ABSCT treatment, toxicities and supportive care

**Fig. 1:**
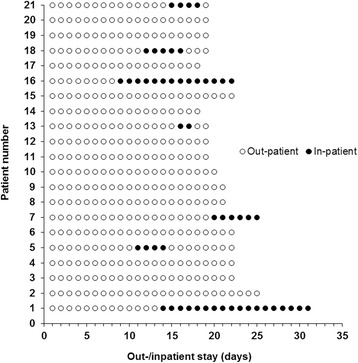
Out- and inpatient stay. Days as out- and inpatient are indicated for each patient. The numerical sequence of the patients (patient number 1 to 21) corresponds to the chronology of the performed ABSCTs

The overall cumulative treatment duration for 21 patients was 444 days, of which 391 days (88%) were spent on an outpatient basis and 53 days (12%) on an inpatient basis. On average, the treatment duration was 21 (range 18–25) and 22 (range 19–31) days for out- and temporary inpatients, respectively. No significant differences in treatment duration were found between the patient cohorts (*P* = 0.38). Overall, 7 patients had an indication for temporary hospital admission. 4 patients were admitted to the hospital because of neutropenic fever persisting more than 72 h (patient no. 5, 7, 18, 21). In 2 further cases, hospital admission was indicated due to grade III stomatitis (patient no. 1 and 16). In one case, inpatient monitoring was initiated due to a local inflammation of the port catheter implantation site (patient no. 13). Patients who were temporarily admitted to the hospital spent a median of 15 (range 8–19) days as outpatients and 5 (range 2–18) days as inpatients. The sequence of days spent as an out- and inpatient during HD chemotherapy and ABSCT for each patient is indicated in Fig. 1. In 3 cases, patients were discharged from the hospital after haematological reconstitution without need for further outpatient treatment.

All patients presented with stomatitis, though to various degrees. Remarkably, only mild grade I stomatitis was observed in the majority of patients (*n* = 17, 81%), and as few as 2 and 2 patients developed grade II and III stomatitis, respectively. Grade III mucositis was defined as a reason for hospital admission.

Red cell and platelet transfusion was performed on 6 patients (29%) and 15 patients (71%) overall, respectively, without significant differences found between the two patient cohorts.

### Infectious complications

Neutropenic fever was observed in 14 patients (67%). In 4 patients, prolonged neutropenic fever longer than 72 h was a reason for hospital admission. 3 patients with neutropenic fever >72 h had only low increase of temperature and were in a good overall condition, so a hospital admission was not initiated (patient no. 2, 15, 19). All of the patients who developed neutropenic fever were treated with i.v. antibiotics (mainly ertapenem 1 mg/d i.v.). In 4 outpatients and 1 inpatient, i.v. antibiotic treatment was initiated due to subfebrile temperatures and CRP elevation.

In 4 patients, the peripheral blood cultures were positive. In one patient (no. 10), *Streptococcus mitis* was detected in peripheral blood culture. An i.v. antibiotic therapy with ertapenem was initiated in this patient on an outpatient basis because the criteria for hospital admission were not fulfilled. In 2 patients, peripheral blood cultures were positive for *Staphylococcus aureus* (patient no. 1) and *Staphylococcus hominis* (patient no. 16), respectively, and a port catheter explantation was performed in these patients due to a suspicion of a port catheter infection. Moreover, *Escherichia coli* was detected in peripheral blood cultures; in this case, inpatient treatment was initiated (patient no. 18). In no case any multi-resistant bacteria were detected. In one patient (no. 7), a port catheter explantation was performed due to persisting fever without any evidence of germs in peripheral or central blood cultures. One patient developed slight diarrhoea, and in one patient, a urinary tract infection was documented. No pulmonary infections and severe adverse events (SAE) were observed. Table 3 gives an overview of the post-ABSCT treatment, toxicities and supportive care provided.

**Table 3:** Post-ABSCT treatment, toxicities and supportive care

Parameters	Overall cohort	Outpatient treatment	Hospital admission	*P*-value
ABSCT number, n	21	14	7	/
Treatment duration				
Overall, days	21 (18–31)	21 (18–25)	22 (19–31)	0.38
Days as outpatient	19 (8–25)	21 (18–25)	15 (8–19)	<0.01
Days as inpatient	0 (0–18)	/	5 (2–18)	<0.01
Reason for hospital admission, n (%)				/
Neutropenic fever ≥72 h	4 (19)	/	4 (57)	
Grade III stomatitis	2 (10)	/	2 (29)	
Other	1 (5)	/	1 (14)	
Toxicities				
Stomatitis, n (%)				
I	17 (81)	13 (93)	4 (57)	
II	2 (10)	1 (7)	1 (14)	
III	2 (10)	0 (0)	2 (29)	
Neutropenic fever				
n, (%)	14 (67)	8 (57)	6 (86)	0.34
No. of days with fever	3 (1–10)	2 (1–7)	4 (2–10)	0.14
Diarrhoea, n (%)	1 (5)	1 (7)	0 (0)	1.00
Pulmonary infection, n (%)	0 (0)	0 (0)	0 (0)	1.00
Urinary tract infection, n (%)	1 (5)	0 (0)	1 (14)	0.33
Positive blood cultures, n (%)				
Peripheral	4 (19)	1 (7)	3 (43)	0.09
Central	0 (0)	0 (0)	0 (0)	1.00
Port catheter infection, n (%)				
Suspicion of	3 (14)	0 (0)	3 (43)	0.03
Proven	0 (0)	0 (0)	0 (0)	1.00
Port catheter explantation	3 (14)	0 (0)	3 (43)	0.03
SAE, n (%)	0 (0)	0 (0)	0 (0)	1.00
Support/Intervention				
Red cell transfusion, n (%)	6 (29)	3 (21)	3 (43)	0.35
Platelet transfusion, n (%)	15 (71)	9 (64)	6 (86)	0.61
i.v. antibiotics				
In case of neutropenic fever, n (%)	14 (67)	8 (57)	6 (86)	0.34
At subfebrile temperature, n (%)	5 (24)	4 (29)	1 (14)	0.62
Overall, days	7 (4–14)	7 (4–10)	8 (4–14)	0.61
Days as outpatient	6 (1–10)	7 (4–10)	1 (0–6)	/
Days as inpatient	5 (2–14)	/	5 (2–14)	/

*ABSCT* autologous blood stem cell transplantation, *i.v*. intravenous, *no*. number, *SAE* severe adverse event. Unless otherwise indicated, data are given as medians (range)

### Hematopoietic reconstitution

The time in aplasia was 11 (range 8–15) and 9 (range 7–11) days in out- and inpatients (*P* = 0.11), respectively. The median time to reach leucocytes ≥1.0 × 10^9^/L after ABSCT was 15 (range 13–20) and 13 (11–16) days for out- and inpatients, respectively. In addition, 14 (range 13–20) and 14 (12–16) days for out- and inpatients were required to reach neutrophil recovery ≥0.5 × 10^9^/L. No significant differences in leucocyte and neutrophil reconstitution were observed between both groups (*P* = 0.11 and *P* = 0.23, respectively). A statistical comparison between the groups in terms of neutrophil recovery was limited by a lack of available neutrophil recovery data.

Because the majority of patients (*n* = 15, 71%) received a platelet transfusion, platelet recovery ≥20 × 10^9^/L could not be evaluated sufficiently. The median number of days to reach platelets ≥50 × 10^9^/L after ABSCT was 14 (range 11–22) in outpatients and 14 (range 11–25) in temporary inpatients. No significant differences in platelet recovery ≥50 × 10^9^/L were observed between both patient cohorts (*P* = 0.97). The hematopoietic reconstitution data after ABSCT are summarized in Table 4. Similar results were observed when leucocyte, neutrophil and platelet reconstitution was analysed as a function of time, using both Kaplan-Meier analysis and log-rank test for curve comparison. No significant differences were found with regard to leucocytes recovery ≥1.0 × 10^9^/L (*P* = 0.14), neutrophil recovery ≥0.5 × 10^9^/L (*P* = 0.33) and platelet recovery ≥50 × 10^9^/L (*P* = 0.59) between the patient cohorts.

**Table 4:** Hematopoietic reconstitution

Parameters	Overall cohort	Outpatient treatment	Hospital admission	*P*-value
Days to L <1.0 × 10^9^/L	4 (2–5)	4 (3–5)	4 (2–5)	0.83
Days to L ≥1.0 × 10^9^/L	14 (11–20)	15 (13–20)	13 (11–16)	0.11
Days in aplasia	10 (7–15)	11 (8–15)	9 (7–11)	0.11
Days to N ≥0.5 × 10^9^/L	14 (12–20)	14 (13–20)	14 (12–16)	0.23
Platelets ≥20 × 10^9^/L				
Platelet transfusion, n (%)	15 (71)	9 (64)	6 (86)	0.61
Analysed ABSCTs, n (%)	6 (29)	5 (36)	1 (14)	/
Days to platelets ≥20 × 10^9^/L	10 (9–16)	10 (9–11)	16	/
Platelets ≥50 × 10^9^/L				
Days to platelets ≥50 × 10^9^/L	14 (11–25)	14 (11–22)	14 (11–25)	0.97

*ABSCT* autologous blood stem cell transplantation, *L* leucocytes, *NA* not available, *N* neutrophils. Unless otherwise indicated, data are given as medians (range)

### Patients’ satisfaction

According to the ratings given by the patients in the questionnaire, the level of satisfaction was high: on a scale from 1 (excellent) to 6 (insufficient), physicians got a rating of 1.1, nurses of 1.2 and the treatment as a whole got a rating of 1.3 (mean values, *n* = 20). All of the patients agreed, if indicated, to undergo further HD chemotherapy and ABSCT in an outpatient setting again. In 3 cases, patients had indeed two consecutive autologous transplants within the program (#6/#18, #8/#10 and #9/#12).

## Discussion

In Europe, HD melphalan chemotherapy followed by ABSCT is performed almost always on an inpatient basis, and only scattered reports on outpatient HD chemotherapy exist [23]. In contrast, in the USA and Canada, outpatient HD chemotherapy and ABSCT in MM and lymphoma patients has been well established for decades [19] and is performed with a high degree of safety [16–18, 26], cost savings [14, 15, 20] and patient satisfaction [13]. As one of the first centres in Europe, we established an outpatient ABSCT program at our institution in 2012. Based on our inpatient HD melphalan chemotherapy and ABSCT treatment protocol, we developed a comprehensive treatment plan for an outpatient setting. Patients were carefully selected and criteria have been developed for hospital admission. Comprehensive patient education about how to behave during aplasia at home took place. Moreover, daily rounds of the outpatients, including vital parameter monitoring, and laboratory tests were performed. The outpatient ABSCT program also included the advanced management of side effects exceeding the standard inpatient care, including a triple anti-emetic regimen, strong recommendation to rinse the mouth with Caphosol® at least once an hour and administration of daily i.v. fluids. Furthermore, with regard to Kim et al., who showed that sequential prophylaxis with oral fluoroquinolone followed by i.v. ertapenem may effectively prevent episodes of bacteremia and hospitalizations in neutropenic MM outpatient ABSCT recipients [27], an empirical i.v. antibiotic therapy was initiated at subfebrile body temperatures when CRP elevation was detected.

Between 2012 and 2016, 21 MM patients underwent HD chemotherapy and ABSCT on an outpatient basis. No SAEs were observed. In our patient cohort, confirmed post-transplant infections were documented in 5 of the 21 patients (24%, positive blood cultures in 4 patients and 1 positive urine culture in 1 patient). This is comparable to the results of Paul et al., who reported an infection rate of 22% (18 of 82 patients) in an initial brief in-hospital stay of MM patients group receiving HD melphalan and ABSCT [17], and to Graff et al., who described an infection rate of 19% (19 of 95 patients) in MM and lymphoma patients undergoing this therapy as outpatients [18]. Less than 10% of patients (2 of 21) developed grade 3 stomatitis. No grade 4 or 5 stomatitis cases were observed. In contrast, Jagannath et al. reported a stomatitis grade ≥3 in 31% of 118 MM patients undergoing outpatient HD chemotherapy and ABSCT [15]. We attribute the low mucositis rate in our patient cohort to regular Caphosol® mouth rinse. Neutropenic fever was observed in two-thirds of the cases. However, the median fever duration was relatively short (2 and 4 days for outpatients and those who required a hospital admission, respectively), and the majority of patients with neutropenic fever (8 of 14) were not admitted for inpatient stay. The neutropenic fever rate is comparable to those observed by Jagannath et al. (50% of 118 outpatient MM auto-transplants [15]) and Leger et al. (56% of 60 outpatient ABSCTs in relapse follicular lymphoma [21]). Moreover, the observed neutropenic fever rate was relatively low compared to in-house historical patient cohorts undergoing HD chemotherapy and ABSCT, with rates of approximately 80% [28, 29]. Although Meisenberg et al. and Paul et al. reported a pulmonary infection rate of 4% (of 27 patients [16]) and 5% (of 82 MM patients [17]) in outpatient auto-transplantation cases, respectively, no pulmonary infections were documented in our patient cohort.

The rate of positive blood cultures in our patient cohort (19%, 4 of 21 patients) is in line with the observation of Graff et al. (10%, 9 of 95 MM and lymphoma ABSCT receiving outpatients [18]) and Paul et al. (16%, 13 of 82 MM patients with an initial in-hospital stay post ABSCT [17]). Moreover, Graff et al. reported 1 central venous line infection among 95 MM/lymphoma patients treated on an outpatient basis (4%) [18]. In our patient group, port explantation was performed in 3 cases (14%) due to a clinical suspicion of port infection upon persisting fever, but without definitive prove of infection by bacterial culture. 90% (19 of 21) of transplanted patients received i.v. antibiotics. Compared to Jagannath et al. who reported a use of i.v. antibiotics in 78% (of 118 MM patients) transplanted in an outpatient setting [15] the higher relative number of patients with i.v. antibiotics in our group can be attributed to an early intervention strategy with initiation of ertapenem infusion at subfebrile temperatures and elevated CRP levels.

Graff et al. observed a neutrophil ≥0.5 × 10^9^/L recovery and platelet ≥20 × 10^9^/L after a median of 10 and 19 days, respectively, in a cohort of MM and lymphoma patients undergoing outpatient ABSCT. We observed neutrophil ≥0.5 × 10^9^/L recovery and platelet recovery after a median of 14 days in both groups, which is almost identical with study data of two historical in-house patient cohorts undergoing HD chemotherapy and ASCT at our institution, with the time to leukocyte increase ≥1 × 10^9^/L and time to platelet increase ≥50 × 10^9^/L being a median of 14 days [28, 29]. Pack red cell and platelet transfusion was necessary in 6 (29%) and 15 (71%) patients. This corresponds to the findings of Jagannath et al. (57% and 97%) [15].

On average, the median treatment duration was 21 and 22 days for outpatients and those who were intermittently admitted to the hospital, respectively. This is in line with the treatment duration of a completely in-hospital-treated MM patient undergoing HD chemotherapy and ABSCT at our institution [29]. Hospital admission was indicated in one-third (7 of 21) of the auto-transplanted MM patients in our cohort. In a comparable MM patient group described by Jagannath et al., 21% of the 118 outpatient transplant procedures required hospital admission [15]. However, in a cohort of 82 MM patients who had an initial brief hospital stay and were followed as outpatients, as described by Paul et al., 67% required hospital re-admission [17]. In our MM patient group, patients who were admitted to the hospital had a relatively short median inpatient treatment of 5 days, and the necessity of hospital admission did not lead to prolonged overall treatment duration. Thus, the temporary inpatient treatment-duration in our cohort was even shorter compared to the cohort of outpatient ABSCTs performed in patients with different hematologic malignancies reported by McDiarmid et al. (median total length of stay 21 d, median inpatient 7 d and median outpatient 14 d) [30].

Overall, approximately 90% (391 days) of the overall cumulative treatment days for 21 patients were spent on an outpatient basis and 10% (53 days) on an inpatient basis. With increasing numbers of outpatient ABSCTs at our centre, the relatively short inpatient stay will represent a significant cost saving option. The magnitude of this effect depends on a number of factors, including reimbursement for in-/outpatient ABSCT, occupancy rate of hospital beds, staff availability etc., and should be addressed in detail in future studies.

Limitations of the presented data result from the relatively small number of outpatients. In addition, this patient cohort was carefully selected and represented only about 5% of all transplanted myeloma-patients at our center during that time period. It therefore represents a pilot-study aiming to proof the feasibility and to describe the necessary preconditions.

According to the results of the structured questionnaire, the patient’s satisfaction with outpatient medical care provided by physicians and nurses as well as their treatment in the outpatient clinic as a whole was very high. In addition, all of the patients indicated willingness to undergo further HD chemotherapy and ABSCT within the outpatient program again, if indicated. This was actually the case in three patients. Further continuation and expansion of the program is intended.

## Conclusions

Carefully selected MM patients undergoing HD chemotherapy and ABSCT can successfully be treated on an outpatient basis with low morbidity and infectious complications and very high patient satisfaction. Although dependent on a number of variables, including the individual compensation agreement with the health-insurance providers, such an approach may also have a significant economic impact on the performing transplant centre.

## Abbreviations

(n)CR: (Near) complete remission
ABSCT: Autologous blood stem cell transplantation
CAD: Cyclophosphamide, doxorubicin, dexamethasone
CRP: C-reactive protein
ECOG: Eastern Cooperative Oncology Group
HD: High-dose
HLA: Human leukocyte antigen
i.v.: Intravenous
L: Leucocytes
MM: Multiple myeloma
MR: Minimal response
N: Neutrophils
NA: Not available
p.o.: Per os
PAD: Bortezomib, doxorubicin, dexamethasone
PBSC: Peripheral blood stem cell
PD: Progressive disease
PR: Partial remission
SAE: Severe adverse events
SD: Stable disease
VAD: Vincristine, doxorubicin, dexamethasone
VCD: Bortezomib, cyclophosphamide, dexamethasone
VGPR: Very good partial remission
